# Crisis social media data labeled for storm-related information and toponym usage

**DOI:** 10.1016/j.dib.2020.105595

**Published:** 2020-04-21

**Authors:** Rob Grace

**Affiliations:** Texas Tech University

**Keywords:** Crisis informatics, volunteered geographic information, Twitter, Information behavior, Emergency management, Risk communication

## Abstract

Social media provides citizens and officials with important sources of information during times of crisis. This data article makes available labeled, storm-related social media data collected over a six-hour period during a severe storm and F1 tornado that struck Central Pennsylvania on May 1^st^, 2017. Three datasets were collected from Twitter using location, keyword, and network filtering techniques, respectively. Only 2% of the 22,706 total tweets overlap among the datasets, providing researchers with a broader scope of information than normally available when collecting tweets using location (i.e., geotag-based) and keyword filtering alone or in combination during a crisis. Each data collection technique is described in detail, including network filtering which collects data from networks of social media users associated with a geographic area.

The datasets are manually labeled for information content and toponym usage. The 22,706 tweet IDs, dehydrated for privacy, are labeled for relevance (storm-related and off-topic) and 19 types of storm-related information organized into six categories: infrastructure damage, service disruption, personal experience, weather updates, weather forecasts, and weather warnings. Data are also labeled for toponym usage (with or without toponyms), location (local, remote, and generic toponyms), and granularity (hyperlocal, municipal, and regional toponyms). The comprehensively labeled datasets provide researchers with opportunities to analyze crisis-related information behaviors and volunteered location information behaviors during a hyperlocal crisis event, as well as develop and evaluate automated filtering, geolocation, and event detection techniques that can aid citizens and crisis responders.

Specifications table

**Table:** 

Subject	Information Science
Specific subject area	Crisis Informatics
Type of data	Datasets
	Tables
How data were acquired	Qualitative content analysis to label Twitter data collected using location, keyword, and network filtering techniques
Data format	Raw
	Labeled
Parameters for data collection	Qualitative content analysis produced six unique sets of labels for tweets posted on Twitter between 3-9pm EST, May 1^st^, 2017
Description of data collection	Tweets were collected using three data collection methods— location, keyword, and network filtering— to gather 22,706 unique tweets during a severe storm and F1 tornado that struck Central Pennsylvania, on May 1^st^, 2017. Location filtering employed Twitter's Public Streaming API to collect 9,109 tweets within a bounding box covering Central Pennsylvania. Keyword filtering employed Twitter's Public Streaming API to collect 4,571 tweets including at least one of 47 place names, including “Centre County,” located in Central Pennsylvania, and the names of Centre County's 46 municipalities, boroughs, and census-designated places. Network filtering employed Twitter's Public Streaming API to collect 8,679 tweets from twitter users with network ties to ground truth accounts within the geographic area of Centre County.
	Qualitative content analysis of the collected tweets involved six rounds of coding to label the data. First, for relevance, tweets were labeled as “storm-related” if referring to the weather or its impacts (e.g., damage caused by high winds, etc.), and “off-topic” if referring to other topics. Second, crisis-related tweets were labeled for 19 types of crisis-related information. Third, storm-related tweets were labeled for six categories of crisis-related information: damages, disruptions, experiences, updates, forecasts, and warnings. Fourth, tweets were coded for toponym usage, distinguishing tweets with specific or generic toponyms from tweets without toponyms. Fifth, tweets with toponyms were coded for references to local places within Centre County, Pennsylvania, references to remote places outside Centre County, and references to generic places, such as “house,” for which a specific location could not be determined. Sixth, tweets with toponyms were further coded for granularity, distinguishing references to hyperlocal places inside cities and counties, municipal places inside intra-state regions and states, and regional places including names of states and other regional location entities.
Data source location	Region: Central Pennsylvania
	Country: USA
Data accessibility	With the article

## Value of the data

• These data are useful for analyzing crisis-related information and volunteered geographic information posted on social media during a crisis.
• Researchers in the fields of crisis informatics and risk and crisis communication can utilize these data to analyze the types of information social media users post during a severe storm. Researchers can also make use of these data to train and evaluate automated filtering, geolocation, and event detection techniques for applications in crisis management.
• Collected using location, keyword, and network filtering techniques and labeled for storm-related information and toponym usage, these data provide more comprehensive and detailed crisis social media datasets than typically available to researchers.
• Additionally, these data provide researchers with opportunities to compare crisis social media gathered with different data collection techniques, opening possibilities for the analysis of “data shadows” [13] cast when using standard location and keyword filtering techniques to collect crisis social media.

## 1 Data Description

The following describes the data available in this article. Supplemental data consists of the *Storm-related Social Media* (SSM) *Dataset* for which the field labels are explained in Table 1 and 2. Additionally, the *User Accounts (UA) Dataset* provides data related to the network filtering technique for which additional data is available in Tables 3 and 4.

**Table 1:** Description of fields and labels in the *Storm-Related Social Media (SSM) Dataset.*

Dataset Field	Description
Tweet ID	Numeric identification number assigned to each tweet by Twitter. In accordance with Twitter's content redistribution policy for academic research [15], only tweet IDs are shared in the *SSM Dataset*. Researchers can “rehydrate” the Tweet IDs through Twitter's API to access the tweet username, text, geographic coordinates, etc.
Collection	Data collection technique employed to filter tweet from Twitter's Streaming API. Labels: *Location, Keyword, Network*
Relevance	Storm-related tweets describe the storm or storm-related impacts while off-topic tweets do not. Labels: *Storm-related, Off-Topic*
Information Type	19 types of storm-related, storm-related information that emerged through the qualitative content analysis. Labels: *Admiration, Advice, Appreciation, Automated Update, Complaint, Current Condition, Event Information, Fear, Flood Advisory, Forecast, Humor, Information Request, Internet Outage, Power Line Damage, Power Outage, Property Damage, Road Damage, Storm Watch/Warning, Tornado Watch/Warning*
Information Category	Six categories organizing the 19 types of storm-related, storm-related information. Labels: *Damages* (power line, property, and road damage), *Disruptions* (power outage, internet outage), *Experiences* (admiration, appreciation, complaint, fear, humor), *Forecasts* (forecast), *Updates* (automated update, current condition, event information, information request), *Warnings* (advice, flood advisory, storm watch/warning, tornado watch/warning)
Toponym Usage	Presence of at least one toponym in the tweet. Labels: *With, Without*
Toponym Location	For tweets with at least one toponym, distinction is made between toponyms referring to locations within or including Centre County, Pennsylvania, toponyms referring to remote locations outside Centre County, and generic toponyms (e.g., house) for which a specific location cannot be determined. Labels: *Local, Remote, Generic*
Toponym Granularity	For tweets with at least one toponym, distinction is made between toponyms referring to hyperlocal places and areas within municipalities, toponyms referring to municipalities withing intra-state regions and states, and toponyms referring to regions within countries.
Time	In accordance with Twitter's content redistribution policy, Tweet IDs are labeled with only quarter hour time stamps for the collection period of 3-9:00 pm EST, May 1^st^, 2017. For example, “3.4” indicates the quarter-hour period 3:45-3:59pm EST.

**Table 2:** Label descriptions for categories and types of storm-related information.

Category	Type	Label Description
Damages	Power Line Damage	Damage to electrical/telephone lines
	Property Damage	Damage to buildings, areas, and infrastructure
	Road Damage	Damage to roads and bridges
Disruptions	Power Outage	Loss or interruption of electrical service
	Internet Outage	Loss or interruption of Internet service
Experiences	Admiration	Awe and wonder for mother nature
	Appreciation	Thanksgiving and respect for first responders
	Complaint	Criticisms of storm impacts on daily life
	Fear	Unease and fright due to storm conditions
	Humor	Comedy, gest, and irony related to storm conditions
Forecasts	Forecast	Predictions of future weather conditions and impacts
Updates	Automated Updates	Weather updates made by automated accounts (bots)
	Current Conditions	Descriptions of current, eyewitness weather conditions
	Event Information	Status updates on community events, e.g., cancellations
	Information Request	Inquiries about weather conditions and events
Warnings	Advice	Weather-related recommendations and directives
	Flood Watch/Advisory	Alert that flooding is possible, imminent, or occurring
	Storm Watch/Warning	Alert that storms are possible, imminent, or occurring
	Tornado Watch/Warning	Alert that tornados are possible, imminent, or occurring

**Table 3:** Place names employed for keyword filtering.

Category	Place Names
County	Centre County
Municipalities	State College, Ferguson Township
Boroughs	Bellefonte, Centre Hall, Howard, Milesburg, Millheim, Philipsburg, Port Matilda, Snow Shoe, Unionville
Census-Designated Places	Aaronsburg, Baileyville, Blanchard, Boalsburg, Clarence, Coburn, Eagleville, Houserville, Hublersburg, Jacksonville, Julian, Lemont, Madisonburg, Mingoville, Monument, Moshannon, Mount Eagle, Nittany, North Philipsburg, Orviston, Park Forest Village, Pine Glen, Pine Grove Mills, Pleasant Gap, Ramblewood, Rebersburg, Sandy Ridge, South Philipsburg, Snydertown, Spring Mills, Stormstown, Toftrees, Woodward, Zion

**Table 4:** Categories and types of community organizations employed for network filtering [4].

Category	Description	# Cataloged
Bars	Establishments of good cheer, e.g. bars, saloons, taverns, wineries, and pubs	27
Citizens’ Associations	Volunteer, social, and non-profit organizations, e.g. habitat for humanity, environmental conservation groups, historical society	41
Civic Services	Civic government and public services, e.g. municipal government, public library, public transit	18
Emergency Services	Emergency management and response services, e.g. police and fire departments, EMS, university emergency alerts	13
Entertainment	Recreational businesses, e.g. minor league baseball team, golf courses, movie theaters	5
Media	Local media, e.g. newspapers, newsletters, news websites, radio, television	30
Restaurants	Local (non-chain) food-serving establishments, e.g. cafes, diners, delis, pizzerias	43
Schools	Municipal school district, e.g. elementary, middle, and high schools, local vocational school	16
Total		193

The *SSM Dataset* includes the three labeled datasets produced through the qualitative content analysis of 22,706 tweets collected via location, keyword, and network filtering during a severe storm and F1 tornado that struck Central Pennsylvania on May 1^st^, 2017. Description of the qualitative content analysis and three data collection techniques are provided in detail in the following section. The *SSM Dataset* includes seven fields which are explained in Table 1.

## 2 Experimental Design, Materials, and Methods

The following sections explain in detail the procedures followed to collect data from Twitter using location, keyword, and network filtering techniques and label the data through six rounds of qualitative content analysis.

### 2.1 Data Collection Procedure

Tweets were collected using three data collection methods— location, keyword, and network filtering— to gather 22,706 unique tweets during a six-hour period (3pm-9am EST) before and after the peak of a severe storm and F1 tornado that struck Centre County, Pennsylvania, just after 6pm EST on May 1^st^, 2017. The collected, labeled data is available in the *SSM Dataset.*

First, location filtering employed Twitter's Streaming API to collect 9,190 tweets with geographic coordinates within a bounding box covering Central Pennsylvania.^1^ Second, keyword filtering employed Twitter's Streaming API to collect 4,571 tweets including at least one of 46 place names, including “Centre County” and the names of the county's 45 municipalities, boroughs, and census-designated places.^2^ The place names employed for keyword filtering are available in Table 3. Third, network filtering involved accessing Twitter's Streaming API to collect 9,026 tweets posted by users with social network ties (i.e. following) to ground truth Twitter accounts associated with Centre County, Pennsylvania. The Twitter account IDs employed for network filtering are available in the *UA Dataset.* As network filtering represents a novel approach to crisis social media collection, additional details about the data collection technique are described below.

Network filtering represents a simple approach to geolocation inferencing. Common geolocation inference methods attempt to infer n-locations for a set of Twitter users, and require “(1) a definition of what constitutes a relationship in Twitter to create the social network, and (2) a source of ground truth location data to use in inference” [7]. Approaches often utilize following or mention network ties among Twitter users [5,16], as well as geographic metadata (i.e., geotags), geographic references in tweet content, or profile location information as the source of ground truth for inferring the locations of other users whose tweets or profiles lack geographic information [5,9]. Lacking external information sources to seed the network with ground truth user locations, these approaches rely on these sparse sources of ground truth data (only 1% of tweets include geotags [11] because they are the only sources available for automated extraction using Twitter's API.

To collect the *SSM Dataset* the geofencing task involved inferring n-users for only a single geographic area (i.e., Centre County), allowing a novel and simplified approach to the geolocation inferencing problem. Exploiting the tendency of local people to follow local organizations [9], network filtering utilizes external information sources to manually determine ground truth accounts whose networks of followers can be inferred as local to the geographic area. This process involves four phases: categorizing ground truth accounts, cataloguing ground truth accounts, collecting user information, and collecting user data.

To categorize ground truth accounts in Centre County, Pennsylvania, a community asset mapping approach was adopted to search for and categorize organizations in a community by type [8,10]. Initial categories of organizations were developed— public institutions, citizens’ associations, local economy (e.g. businesses), and media— which were subsequently modified and expanded by searching for and cataloguing organizations maintaining a Twitter account in the geographic area of interest. The resulting eight categories of community organizations are listed in Table 4.

The second phase, cataloging ground truth accounts, involved identifying a comprehensive list of organizations for each of the eight categories identified in phase one. Online directories and search engines were used to discover organizations and identify associated Twitter accounts. Additionally, Twitter recommendation algorithms were consulted to suggest additional organizations during the search process to identify any organizations that did not appear in online searches. Altogether 195 organizations were catalogued across eight categories. In accordance with Twitter's content redistribution policy for academic research [15], the account IDs are made available in the *UA Dataset*.

The third phase involved use of Twitter's REST API to extract the account IDs of users following the 195 organizations manually identified as ground truth accounts. Altogether, 185,176 Twitter account IDs were extracted. This follower count reflects the period of data collection, which occurred during the week of December 5th, 2016. Researchers can use the *UA Dataset* to reproduce the procedure.

Lastly, the fourth phase involves utilizing the extracted account IDs and Twittter's Streaming API to collect tweets the networks of users following the ground truth accounts. Using this network filtering technique, data was collected during the 6-hour period on May 1^st^, 2017 to produce the *SSM Dataset*. Interested researchers can consult studies of Grace et al. [3,4] that evaluate the accuracy of this “wide net” inferencing approach and nature of information Twitter users posted during the storm.

### 2.2 Data Labeling Procedure

The 22,706 tweets were manually assigned six sets of labels produced through a qualitative content analysis. First, to determine relevance, tweets were coded as “storm-related” if referring to weather or its consequences (e.g., damage caused by high winds, etc.), and “off-topic” if referring to other topics (e.g., sporting events, political issues, etc.). A random set of 1000 tweets were coded by three researchers and a Cronbach alpha test was run yielding α = 0.92. Coding differences were deliberated and reconciled, and then the remaining data was subdivided and coded for relevance.

Second, storm-related tweets were labeled for information type. Together, the three authors engaged in a grounded, iterative process of open coding to label all storm-related tweets [1,^2^,6]. During this process the authors consulted and extended codes developed in previous studies [12,14]. For what Olteanu et al. [12] label as “Infrastructure & Utilities,” for example, five new labels emerged in the data: property damage, road damage, power line damage, Internet outage, and power outage. Ultimately, the coding process resulted in 19 types of information which are described in Table 2. The remaining labelling process was performed individually by the author.

Third, storm-related tweets were labeled for six categories of crisis-related information. These categories organized related types of information together resulting in the higher-level labels that provide researchers with opportunities to examine information posted during the storm at different levels of detail. The categories and associated information types (in parentheses) include: damages (power line, property, and road damage), disruptions (power outage, internet outage), experiences (admiration, appreciation, complaint, fear, humor), forecasts (forecast), updates (automated update, current condition, event information, information request), and warnings (advice, flood advisory, storm watch/warning, tornado watch/warning).

Fourth, tweets were coded for toponym usage, distinguishing tweets with specific or generic toponyms from tweets without toponyms. Toponyms are place names which can be defined as “specific,” referring to a unique place (e.g., 126 Main Street), or “generic,” referring to a class of place or instance of that class (e.g., that house). Any tweet with at least one toponym, whether specific or generic, was coded as “with” toponyms.

Fifth, tweets with toponyms were coded for location reference. Toponyms referring to places within Centre County, Pennsylvania were labeled “local.” Toponym references to places outside Centre County were labeled as “remote.” Toponym references to generic places, such as “house,” for which a specific location could not be determined, were labeled “generic.” If a tweet contained both specific and generic toponyms, the tweet was labeled according to the location reference of the specific toponym.

Sixth, and lastly, tweets with toponyms were coded for granularity. Toponyms referring to places and areas inside cities and counties were labeled “hyperlocal.” Toponyms referring to towns, cities, and counties inside inside intra-state regions and states were labeled “municipal.” Toponyms referring to intra and inter-state regions and states were labeled “regional.”

## 3 Ethical Issue

In accordance with Twitter's Developer Policy [15], these data include only tweet IDs and user IDs removed of all tweet content, metadata, and personally identifying information. Twitter's terms for “content redistribution” stipulate that:

If you provide Twitter Content to third parties, including downloadable datasets or via an API, you may only distribute Tweet IDs, Direct Message IDs, and/or User IDs (except as described below). We also grant special permissions to academic researchers sharing Tweet IDs and User IDs for non-commercial research purposes…Academic researchers are permitted to distribute an unlimited number of Tweet IDs and/or User IDs if they are doing so on behalf of an academic institution and for the sole purpose of non-commercial research. For example, you are permitted to share an unlimited number of Tweet IDs for the purpose of enabling peer review or validation of your research. [15]

The *SSM Dataset* and *UA Dataset* distributed with this article include only user and account IDs. These data are distributed for the sole purpose of non-commercial research in strict compliance with Twitter's Developer Policy.

## Appendix Supplementary materials

Application 1

Application 2

## Footnotes

1 SW 40.170196, -79.1214656; NW 41.3434653, -76.5996462.

2 http://centrecountypa.gov/.
